# Stem Cell Interaction with Somatic Niche May Hold the Key to Fertility Restoration in Cancer Patients

**DOI:** 10.1155/2012/921082

**Published:** 2012-04-02

**Authors:** Deepa Bhartiya, Kalpana Sriraman, Seema Parte

**Affiliations:** Stem Cell Biology Department, National Institute for Research in Reproductive Health, Parel, Mumbai 400 012, India

## Abstract

The spontaneous return of fertility after bone marrow transplantation or heterotopic grafting of cryopreserved ovarian cortical tissue has surprised many, and a possible link with stem cells has been proposed. We have reviewed the available literature on ovarian stem cells in adult mammalian ovaries and presented a model that proposes that the ovary harbors two distinct populations of stem cells, namely, pluripotent, quiescent, very small embryonic-like stem cells (VSELs), and slightly larger “progenitor” ovarian germ stem cells (OGSCs). Besides compromising the somatic niche, oncotherapy destroys OGSCs since, like tumor cells, they are actively dividing; however VSELs persist since they are relatively quiescent. BMT or transplanted ovarian cortical tissue may help rejuvenate the ovarian niche, which possibly supports differentiation of persisting VSELs resulting in neo-oogenesis and follicular development responsible for successful pregnancies. Postnatal oogenesis in mammalian ovary from VSELs may be exploited for fertility restoration in cancer survivors including those who were earlier deprived of gametes and/or gonadal tissue cryopreservation options.

## 1. Introduction

Stem cells hold tremendous potential and promise for regenerative medicine and have raised the hope of the public for a cure for several diseases. Reproductive biologists and infertile couples are further excited by the concept of deriving “synthetic gametes” from pluripotent stem cells, but one wonders whether generation of “synthetic gametes” is more of science fiction or a realistic option for healthy babies in the future. Hübner et al. [1] were the first to report spontaneous generation of oocytes enclosed within structures that resembled developing ovarian follicles by differentiation of mouse embryonic stem cells *in vitro*. Fetal pig skin stem cells [2] and rat pancreatic stem cells [3] cultured *in vitro* have also been shown to generate oocyte/follicle-like structures. Daley [4] summarized that the development of synthetic gametes from embryonic stem cells is fascinating basic research, but the clinical application is still a hypothetical possibility. The idea of producing gametes from induced pluripotent stem cells derived from skin fibroblasts has also been proposed [5]. Although interesting, the major concern that would limit translation of these research efforts into clinical applications is epigenetic and genetic stability of the gametes produced [6]. The other challenge involves establishing protocols to achieve robust and functional oocyte differentiation from embryonic stem cells, and at present this remains a highly inefficient process.

Another fast expanding area is the presence of stem cells in adult mammalian ovaries. The mammalian ovary harbors stem cells and possibly undergoes postnatal oogenesis during reproductive life rather than being endowed with a finite pool of primordial follicles at birth. Johnson et al. [7] provided evidence in support of postnatal oogenesis and challenged the six-decade-old paradigm by conducting simple experiments. The group demonstrated using mouse ovary the rate at which follicular atresia occurs, the ovary should be devoid of follicles by young adulthood, but this never happens. However, the idea of the ovary harboring stem cells is still not well accepted amongst reproductive biologists, and Notarianni [8] have recently reviewed available data in support and against the presence of stem cells in the postnatal ovary. Research in the area of germ line stem cells in mice as well as human ovaries by various groups has recently been elaborately reviewed [9]. 

One of the markers to identify stem cells is OCT-4 (Pou5f1), an octamer binding nuclear transcription factor. It is normally used to define pluripotent state of a stem cell and is well studied in embryonic (ES) and carcinoma stem cells. It is also a germline-specific maternally expressed factor [10]. During embryonic development, OCT-4 is expressed by primordial germ cells (PGCs) and germ cells. Recently OCT-4 positive pluripotent very small embryonic-like stem cells (VSELs) have been reported in various adult somatic tissues including bone marrow and cord blood in mice as well as humans [11–13]. OCT-4 biology has indeed surprised and confused stem cell biologists due to the existence of its isoforms [14–16]. The pluripotent stem cell properties of OCT-4 are because of OCT-4A isoform localized in the nuclei as a transcription factor, whereas Oct-4B isoform is localized in the cytoplasm and has no known biological function [17, 18]. We recently reported nuclear OCT-4A positive VSELs in adult human and mice testis [19, 20]. These cells possibly undergo asymmetric cell division to give rise to slightly bigger A_dark_ spermatogonial stem cells (SSCs), which have cytoplasmic OCT-4B. OCT-4 expression is lost as the testicular germ cells undergo further differentiation and meiosis. Similar stem cell biology also exists in the adult mammalian ovary, which will be explained in the subsequent sections. Unlike testis, OCT-4 continues to be expressed in growing follicles, since it is a maternally inherited gene but this will not be elaborated further as it is beyond the scope of this paper.

## 2. Stem Cells in Ovaries

Mitotically active germ cells expressing mouse VASA homolog (MVH) and synaptonemal complex protein 3 (SCP3) were reported in the adult mouse ovarian surface epithelium (OSE) by Johnson et al. [7]. Niikura et al. [21] reported that aged mouse ovaries possess premeiotic germ cells that differentiate into oocytes on transfer into a young ovarian environment. Recently Zou and coworkers used MVH and FRAGILIS-based sorting method to isolate female germ line stem cells (FGSCs) from mouse ovaries [22, 23]. The MVH-sorted FGSCs of about 10–12 *μ*m were cultured for more than 15 months and on transplantation in busulfan-treated mice resulted in live-births demonstrating postnatal oogenesis. Pacchiarotti et al. [24] have demonstrated the presence of FGSCs in postnatal mouse ovary using transgenic mice that express green fluorescent protein (GFP) under the control of Oct-4 promoter. They reported three different types of GFP-OCT-4 positive cells based on size—small (10–15 *μ*m) sized cells in the ovarian surface epithelium, medium (20–30 *μ*m) and big (50–60 *μ*m) oocytes in the follicles. Ploidy analysis based on flow cytometry showed that 70% of these cells were tetraploid oocytes and 30% were diploid stem cells. Gong et al. [25] derived two pluripotent colony-forming cell lines from adult ovarian stromal cells, which also formed embryoid bodies and teratomas. They concluded that embryonic-like stem cells exist in either the ovarian stroma or the stromal cells, get reprogrammed *in vitro* to embryonic-like state. They have also reported that a small subgroup of the dissociated cells from adult ovary (unlike spleen and small intestine) is immunoreactive for both OCT-4 and NANOG (pluripotent marker). Reverse transcription-PCR (RT-PCR) results also demonstrate the presence of transcripts for both Oct-4 and Nanog in adult ovarian tissue. 

**Figure 1:**
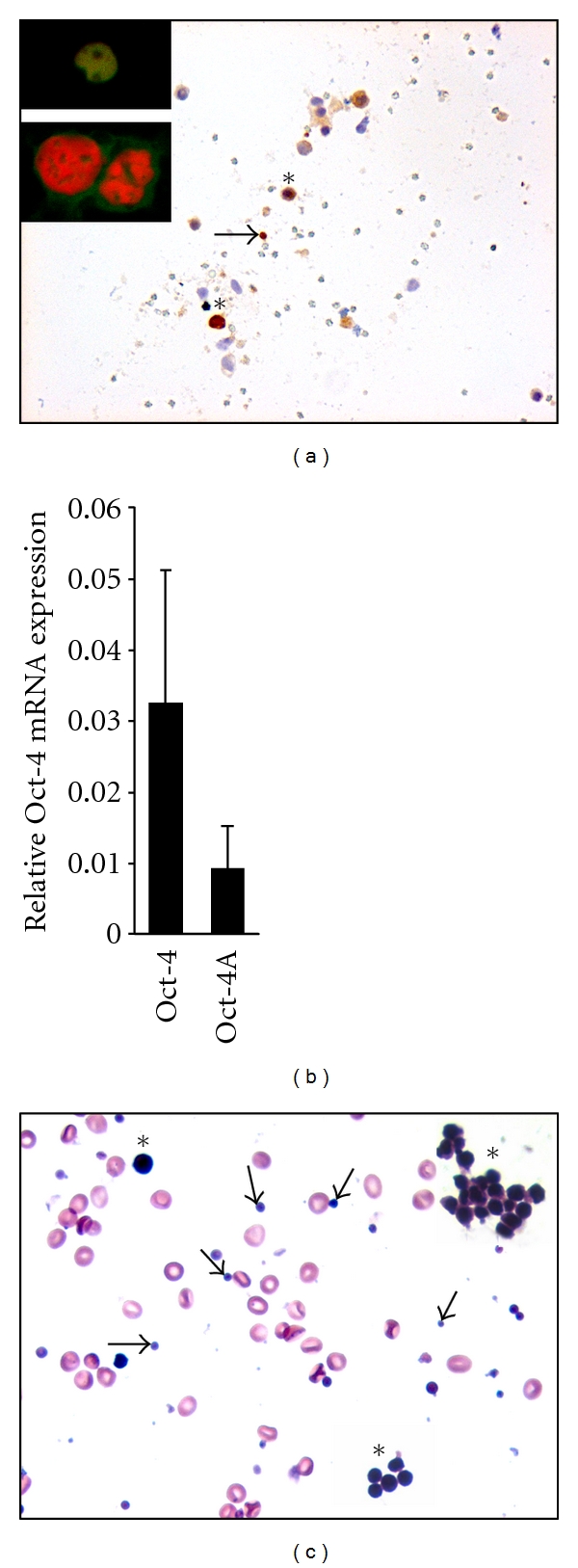
VSELs and OGSCs in adult mammalian ovary. (a) Immunolocalization of OCT-4, a stem cell marker on mouse ovarian cell smear using polyclonal antibody raised against C-terminal domain of OCT-4 (magnification 20x). Two distinct populations of stem cells were observed nuclear OCT-4 positive VSELs (arrow) and cytoplasmic OCT-4 positive OGSCs (asterisk). Inset is representative of the two stem cell populations by confocal microscopy using propidium iodide (PI) as a counterstain (magnification 63x with 5x optical zoom). VSEL has yellow stained nuclei as a result of co-localization of FITC labeled OCT-4 and PI whereas OGSC has distinct PI-stained red nuclei and cytoplasmic OCT-4. (b) Relative expression of Oct-4 and Oct-4A (transcript specific for pluripotent state) mRNA levels in normal mouse ovary by Q-PCR analysis. The levels of Oct-4A transcript in comparison to total Oct-4 were significantly lower suggesting that the VSELs positive for Oct-4A are less abundant compared to OGSCs. (c) H & E staining of human perimenopausal ovary surface epithelium smear showing the presence of RBCs, very small VSELs (arrow), and slightly bigger OGSCs (asterisk; present either as isolated cells or as clusters termed “germ cell nests” in developing ovary) (magnification 40x). Note the high nucleo-cytoplasmic ratio in stem cells with intense nuclear Hematoxylin staining.

Studies on human ovarian stem cells are relatively few in number because of scarcity of the ovarian tissue for research. Bukovsky et al. [26] were the first to show that scraped surface epithelium of postmenopausal human ovary develops into oocyte-like structures of about 180 *μ*m in the presence of a medium with phenol red (estrogenic stimuli). Virant-Klun and her group [27–29] identified putative stem cells in ovarian sections and also in scraped ovarian surface epithelium (OSE) of postmenopausal women and those with premature ovarian failure. These stem cells express pluripotent transcripts Oct-4, Sox2, and Nanog, expressed cell surface antigen SSEA4, and differentiated into oocyte-like structures and parthenotes *in vitro*. We have recently shown the presence of VSELs in ovaries which can be easily isolated by gentle scraping of OSE in adult rabbit, sheep, monkey, and perimenopausal women. These stem cells spontaneously differentiate into oocyte-like structures and parthenotes *in vitro* [30] in agreement with published literature [28, 29, 31]. Besides VSELs with nuclear OCT-4, we have also shown slightly larger cells with cytoplasmic OCT-4, termed ovarian germ stem cells (OGSCs) similar to the terminology used by Pacchiarotti's group. Similar to testis, VSELs with nuclear OCT-4A are relatively less in numbers in ovary as compared to the progenitors (OGSCs) with cytoplasmic OCT-4B. Similarly, two distinct populations of stem cells were also detected in adult mouse ovaries by immunolocalization and quantitative PCR (Q-PCR) analysis (Figure 1). Nuclear Oct-4A transcripts are less abundant as compared to total Oct-4 transcripts that include both A and B isoforms. Thus probably a similar pluripotent stem cell network exists in the gonads of both sexes in mice as well as humans.

The VSELs are probably the PGCs persisting into adulthood as suggested by others as well [32, 33]. Ratajczak and his group were the first to report presence of VSELs in adult body tissues and have made significant contribution in the field, which was recently compiled [33]. It is believed that a common VSEL stem cell population exists in various body tissues, and depending on its immediate microenvironment, they differentiate into that particular lineage [11]. VSELs are highly mobile in nature, and whenever there is any damage or disease in any part of the body, they get mobilized into circulation from the bone marrow [34–37]. 

At this juncture, it becomes crucial to comprehend and consolidate the various published studies so that a strong and clear concept emerges.  Table 1 is a list of various publications on ovarian stem cells and our attempt to explain the results in the context of VSELs biology. As evident, there is a general agreement in the location of ovarian stem cells in the OSE.

## 3. Proposed Model for Oogenesis and Follicular Assembly in Adult Mammalian Ovary

Ovary harbors two distinct populations of stem cells, namely, VSELs and OGSCs (Figure 1). VSELs are quiescent stem cells whereas OGSCs are the progenitor stem cells, which proliferate, form germ cell nests, and differentiate into oocytes that get surrounded by somatic cells and assemble into primordial follicles. This model comprising two distinct stem cell populations in the gonads is in agreement with the concept put forth by Li and Clevers [38] in various adult body tissues like bone marrow, hair, and gut epithelium. Like the A_dark_ SSCs in the testis, OGSCs in the ovaries also have a relatively dark nucleus after Hematoxylin and Eosin (H & E) staining. This possibly reflects simple stem cell biology *in vivo* wherein the open euchromatin of pluripotent VSELs possibly gets compacted, appears dark, and undergoes remodeling and reprogramming for differentiation into a particular lineage. During “nuclear reprogramming” a dramatic change in facultative heterochromatin occurs [39]. Cells with pluripotent properties, that is, the nuclear Oct-4A positive cells, probably have abundant transcription permissive euchromatin, which becomes compacted due to stable association of histones with the chromatin in A_dark_ SSCs in testis and OGSCs in ovary, similar to that reported during ES cell differentiation [40]. Thus, because of intense “nuclear reprogramming” the early progenitor cells, namely, OGSCs and A_dark_ SSCs appear dark.

**Figure 2:**
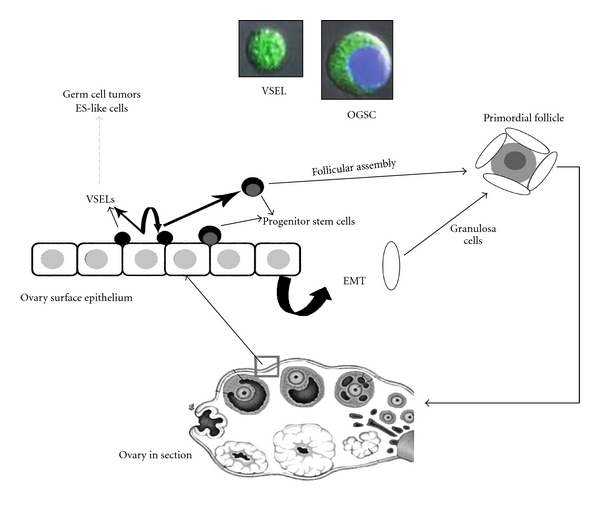
Proposed model for postnatal oogenesis in adult mammalian ovary. Pluripotent stem cells with nuclear OCT-4 (VSELs) being located in the ovary surface epithelium (OSE). These cells undergo asymmetric cell division and give rise to cells with cytoplasmic OCT-4 (OGSCs, which intensely stain with Haematoxylin). The OGSCs undergo further proliferation, meiosis, and differentiation to assemble into primordial follicles in the OSE. The granulosa cells are formed by the epithelial cells that undergo epithelial mesenchymal transition [30]. As the follicles grow and further mature, they shift into the ovarian medulla. Confocal images represent VSEL and OGSC isolated by scraping the surface epithelium of perimenopausal human ovary [30].

During three-week culture of the scraped OSE cells, the stem cells give rise to oocyte-like structures whereas the epithelial cells undergo epithelial-mesenchymal transition (EMT) to give rise to somatic granulosa-like cells [30]. The granulosa-like cells surround the developing oocyte resulting in follicular assembly *in vitro*. The differentiating oocyte undergoes meiosis and exhibits various germ cell markers, formation of Balbiani body-like structures, and characteristic cytoplasmic streaming *in vitro* (unpublished data). Similar views have been recently put forth by other groups as well [41, 42]. Bukovsky and group have proposed that possibly this EMT *in vivo* occurs in the tunica albuginea region of the ovary and may be involved in primordial follicle assembly. Figure 2 is a diagrammatic representation of the proposed model for postnatal oogenesis and follicular assembly from ovarian stem cells. 

## 4. Stem Cells, Somatic Niche, and Menopause

Menopause implies exhausted ovarian follicle reserve and may be age related or induced prematurely by gonadotoxic insults including oncotherapy in the case of cancer survivors. But several groups have shown the presence of stem cells in the OSE of postmenopausal ovary [27–30] and in aged mouse ovary [21]. Why are these stem cells unable to differentiate and replenish the follicular pool? Why does menopause occur? The emerging literature supports the concept that it is most likely a compromised somatic niche (a cellular and molecular microenvironment that regulates stem cell function) that is unable to support stem cell differentiation [41, 43, 44] that causes menopause. Niikura et al. [21] demonstrated that stem cells exist in aged ovary, which is otherwise devoid of any oocytes. To demonstrate that the stem cells still retain the differentiation potential, they performed ovarian transplantation studies. Grafting of aged ovarian tissue of Oct4-GFP transgenic mice onto wild type young mouse ovary resulted in follicles containing GFP positive oocytes. In contrast, exposure of young ovarian tissue to aged environment resulted in reduced number of immature follicles. They proposed that failure of oocyte replenishment in the aged ovary was probably due to impairment of the somatic microenvironment rather than depletion/aging of the stem cells. In a similar study performed previously in male mice, SSC transplantation in irradiated testis was only able to support colonization and not differentiation. This has lead to a similar conclusion that the compromised somatic niche does not support stem cell differentiation [45]. 

We studied the presence of VSELs and OGSCs in chemo-sterilized mouse ovaries. We have observed that the quiescent VSELs persist and are resistant to therapy whereas the rapidly dividing OGSCs and mature follicles are lost resulting in premature ovarian failure (unpublished results). Similar resistance of VSELs has recently been demonstrated in mouse bone marrow after whole body irradiation [46].

It becomes pertinent to refer to two published studies here. Firstly Lee et al. [47] could rescue chemotherapy-induced premature ovarian failure in a mouse model by bone marrow (BM) transplantation. They were however intrigued by the fact that all the pregnancies were of recipient origin and not of donor BM. Their results can be explained, if we consider that the autologous VSELs that survived chemotherapy (because of their quiescent nature) underwent differentiation, folliculogenesis, and pregnancy in response to some signal provided by the transplanted BM. Secondly Fu et al. [48] transplanted bone-marrow-derived mesenchymal stem cells (MSCs) in ovaries of chemotherapy-induced ovarian damage and reported improved ovarian function. They showed that the MSCs secreted cytokines and inhibited chemotherapy-induced apoptosis of granulosa cells. They concluded that transplanted MSCs play an important role in ovarian microenvironment and protect ovary from chemotherapy-induced damage through secretion of cytoprotective proteins.

## 5. Clinical Evidence for Spontaneous Restoration of Fertility

The current available options offered to female cancer patients for fertility preservation include gonadal shielding, cryopreservation of egg/embryo, and/or ovarian cortical tissue prior to oncotherapy. The eggs or embryos are utilized to achieve parenthood by standard assisted reproductive techniques when required whereas the cryopreserved ovarian tissue fragments are transplanted at either orthotopic or heterotopic site to serve as a source of gametes [49]. To date thirteen pregnancies have been reported after orthotopic transplantation of ovarian cortical tissue on the surface of the atrophied ovary [50]. Interestingly, after heterotopic transplantation of cryopreserved ovarian cortical tissue or after allogeneic bone marrow transplantation, spontaneous recovery of intact, atrophied, and menopausal ovary has been reported resulting in spontaneous pregnancies [51–55]. Similarly a study has shown that bone marrow transplantation (BMT) in aged mice also helps sustain ovarian function [56]. 

Fertility restoration in these cases could be because of (i) restoration of lost germ stem cells or (ii) improved functionality of compromised niche in the atrophied ovary that is now able to support oogenesis and follicular assembly. Veitia et al. [57] provided evidence that spontaneous fertility after oncotherapy or allogeneic bone marrow transplantation was not because of donor bone-marrow-derived germline stem cells, as microsatellite analysis showed that the baby was of recipient origin. Thus it is becoming clear that the BMT or transplanted tissue somehow provides the necessary endocrine/paracrine signals to the compromised niche (rather than being a source of oocytes) and helps in restoration of ovarian function. The stem cell connection with spontaneous restoration of fertility has already been suggested by Oktay [58].

Research efforts must be intensified to identify the actual factors that are essential to restore functionality of the gonadal niche. Similar regenerative signals exist in young and aged male blood [59] which can also rejuvenate follicular dynamics in an aged ovary. Sönmezer et al. [60] have thrown open a discussion that low levels of androgens may have a role in the regenerative effect reported by Niikura et al. [59]. To support their view they gave the example of polycystic ovarian syndrome, where mildly increased androgens may be responsible for higher than average number of follicles observed and delayed menopause. Whether it is a reflection of increased stem cell activity needs to be demonstrated! Secondly treatment with dehydroepiandrosterone (a mild androgen) has been shown to improve ovarian response to fertility drugs [60].

## 6. Conclusion

This paper consolidates the published literature and discusses it in the context of the existence of two distinct stem cell populations in the ovary in an effort to bring more clarity in the field of adult mammalian oogenesis. It also discusses the possibility of restoring fertility by reconstructing the ovarian somatic niche. If true, various epigenetic and genetic concerns associated with long-term culture and differentiation of embryonic stem cells to make “synthetic gametes” or *in vitro* culture of OSE to generate autologous oocytes or maturation of primordial follicles *in vitro* may be overcome. This approach will open up new and novel, non-invasive avenues for fertility restoration, offer new means to treat female infertility, and delay menopause. Moreover, even patients who were deprived of fertility preservation options prior to oncotherapy stand to benefit by advances in this field.

## Figures and Tables

**Table 1:** Consolidation of published literature on stem cells in adult mammalian ovary based on the concept of pluripotent (VSELs) and progenitor stem cell population (OGSCs).

Reference	Study highlights	Interpretation of published literature
Johnson et al. [7] Germline stem cells and follicular renewal in the postnatal mammalian ovary	Mitotically active SCP3^+^ and MVH^+^ germline stem cells in surface epithelium of adult mice ovary Chimeric follicles observed when wild type ovarian tissue is grafted onto ovary of GFP expressing transgenic mice	Several groups including our results also report the presence of stem cells in the ovary surface epithelium Probably they detected the bigger OGSCs since the cells were SCP3^+^ and MVH^+^ Chimeric follicles suggest that the oocyte and granulosa cells do not originate from a common bipotent progenitor stem cell as suggested by Bukovsky et al. [26, 61, 62]

Johnson et al. [63] Oocyte generation in adult mammalian ovaries by putative germ cells derived from bone marrow and peripheral blood	Extraovarian bone marrow (BM) origin of germ stem cells (GSCs) in adult mice and distribution by peripheral blood (PB) to the ovaries BM and PB express primordial germ cell markers Oct-4, Dazl, Mvh, Stella, Fragilis, and Nobox	Compromised ovaries in young mice (due to chemotherapy) possibly mobilized VSELs from the bone marrow to enter circulation In addition to the pluripotent markers (Oct-4 and Nanog), the mobilized VSELs expressed germ cell specific markers. On similar note during stroke the mobilized VSELs exhibit neural markers like GFAP, nestin, beta-III-tubulin, Olig1, Olig2, Sox-2, and Musashi [35] Possibly the VSELs sense the nature of damage—and thus proliferate and give rise to progenitor stem cells exhibiting specific markers Interestingly the mobilized cells reported were Lin^−^ and Sca^−^, implying that they neither were of hematopoietic origin nor were pluripotent. The authors probably detected GSCs which expressed germ cell specific markers

Lee et al. [47] Bone marrow transplantation generates immature oocytes and rescues long-term fertility in a preclinical mouse model of chemotherapy induced premature ovarian failure	Chemotherapy sterilized mice were transplanted BM cells from coat color mismatched donors All pups born were of recipient germ line	BMT possibly provides an endocrine/paracrine signal that improves the functionality of ovarian niche there by restoring function BM does not serve as a source of germ cells since all the pups are similar to the recipient

Bukovsky et al. [64] Bone-marrow-derived cells and alternative pathways of oogenesis in adult rodents	Suggested alternative pathway of oogenesis in adult rodents Explained that the rodent germ cells may, but do not necessarily originate from the OSE stem cells. Proposed alternative origin of putative germ cells from the medullary region Used neonatally estrogenized female rats which lack OSE but with normal stock of primordial follicles as study model Showed clusters of SSEA1^+^ cells in the ovarian medulla-precursors of oocytes Proposed that female germ cells should receive an impulse from the immune system-related cells to become oocytes. Therefore, if triggered by BM derived cells, the germ cells in ovarian medulla may represent an alternative source of oocytes for renewal of primary follicles	We propose that there may not be any alternative pathway existing in rat ovaries Ovary after neonatal exposure to estradiol is compromised and its homeostasis is disturbed. This may mobilize VSELs from BM through PB. Mobilized VSELs possibly enter ovarian medulla through the blood vessels and then try to reach the cortex for follicular assembly Their results probably show that SSEA1^+^ cells migrate from the BM into the medulla of the ovaries

Szotek et al. [65] Normal ovarian surface epithelial label-retaining cells exhibit stem/progenitor cell characteristics	Identified a label-retaining cell (LRCs) population in coelomic epithelium of adult H2B-GFP transgenic mouse ovary These cells exhibit quiescence, functional response to the estrus cycle, slow cycling, and may undergo asymmetric cell division, exhibit cytoprotective mechanisms by enrichment for side population, and show increased growth potential *in vitro *	The LRCs reported by them are possibly the VSELs which undergo asymmetric cell division Interestingly VSELs do not stain with DAPI possibly because they mostly comprise of euchromatin whereas DAPI binds preferentially to heterochromatin [30]

Zhang et al. [66] Expression of stem and germ cell markers within nonfollicle structures in adult mouse ovary	Used Oct-4-EGFP transgenic mouse model to study the expression of stem and germ cell markers in adult murine ovaries OCT3/4, MVH, SSEA-1, and SCF-R in specific cell aggregates of 50–200 cells (distinct from follicles) within the adult mouse ovary Aggregates have large round nuclei, intensely stain with Haematoxylin, positive for OCT-4, SSEA-1, SCF-R, and MVH; also have SCP3 and DMC1 (meiotic markers by RT-PCR); interestingly they lacked GDF-9 (a postmeiotic marker) Authors conclude a mixed population of committed stem cells as well as transitional stage germline cells that might retain the capacity of proliferation and differentiation	These aggregates possibly represent clonal expansion of OGSCs with cytoplasmic continuity described as germ cell nests in developing fetal ovary [67]. We have observed similar structures in adult mouse and human ovary (Figure 1). Like the OGSCs, cells comprising the germ cell nests have characteristic dark stained nuclei after H staining. OGSCs are immediate progenitors of VSELs and since this involves a shift from euchromatin to a committed genome of a germ cell—extensive chromatin compaction, remodeling occurs—giving dark appearance after H stain. OGSCs divide rapidly to form germ cell nests. This data directly supports postnatal oogenesis in adult mammalian ovary. However, the group have reported OGSCs and not VSELs

Zou et al. [22] Production of offspring from a germline stem cell line derived from neonatal ovaries	Proliferative MVH positive (10–12 *μ*m) large FGSCs purified from neonatal and adult mouse ovaries and maintained *in vitro* for months These cells, after transplantation into ovaries of chemotherapy sterilized recipients, generate chimeric follicles that were fertilized and produced viable offspring	Such large VASA positive cells have been reported also by Zhang et al. [66] However, they immunosorted the initial cells for establishing the cultures based on MVH, a germ cell marker and not an early stem cell marker

Zou et al. [23] Improved efficiency of female germline stem cell purification using fragilis-based magnetic bead sorting	Use of Fragilis, an early germ cell marker, to enrich cells (10–12 *μ*m) for initiating cultures—further enhanced isolation efficiency of mouse FGSCs	Possibly sorted OGSCs based on the size of the cells sorted by them

Pacchiarotti et al. [24] Differentiation potential of germ line stem cells derived from the postnatal mouse ovary	Demonstrated the presence of GSCs in adult mouse ovary using Oct-4-EGFP transgenic mouse model Detected three different types of GFP-OCT-4 positive cells based on size, namely, small (10–15 *μ*m) sized in OSE; medium (20–30 *μ*m) and big (50–60 *μ*m) oocytes in the follicles by flow cytometry Ploidy analysis results showed that 70% of these cells were tetraploid (possibly oocytes) and 30% were diploid (stem cells). They further showed that CD133^+^ cells exist in the ovary but do not co-localize with GFP-OCT-4 suggesting that germ line stem cells in ovary are distinct from the circulating CD133^+^ cells	Flow Cytometry data shows that diploid stem cells exist in ovary and are further of two sizes in agreement with our data However, their approach of using GFP-OCT-4 mice did not allow them to differentiate between cytoplasmic and nuclear OCT-4 since GFP will be expressed by both the stem cells as both the transcripts are under the control of common Oct-4 promoter. CD133^+^ cells are possibly the VSELs but it is intriguing that they did not co-express OCT-4 Thus whether nuclear OCT-4 positive VSELs express GFP or not needs further investigation

Gong et al. [25] Embryonic stem cell-like cells established by culture of adult ovarian cells in mice	Ovarian stromal cells (<40 *μ*m) were subcultured on fibroblast monolayer and colony-forming cells were characterized Detected pluripotent stem cells in adult mice ovary which could be expanded in culture Two ES-like cell lines were established which expressed pluripotent markers and formed embryoid bodies and teratomas	The group was unable to provide information on the exact location on the pluripotent stem cells since they used all cells of size less than 40 *μ*m to establish cultures. They mention stromal origin of stem cells but cells for initiating cultures were obtained by mincing whole ovary which will include the OSE also Ovarian smears used to demonstrate the presence of Oct-4 positive cells and also RNA was extracted from the whole ovary for RT-PCR to show the pluripotent transcripts—thus OSE as a source of pluripotent stem cells is not ruled out in their study

Bukovsky et al. [26, 61, 62] Immunohistochemical studies of the adult human ovary: possible contribution of immune and epithelial factors to folliculogenesis Origin of germ cells and formation of new primary follicles in adult human ovaries Oogenesis in cultures derived from adult human ovaries	Putative germ cells within the OSE of adult human ovary and originate from OSE stem cells which differentiate from mesenchymal progenitors in the ovarian tunica albuginea Scraped OSE cells from adult human ovary in culture form large oocyte-like cells and follicle-like structures Put forth the concept of bipotent progenitors capable of differentiating into oocytes and granulosa cells	The model of bipotent progenitors giving rise to germ and granulosa cells does not explain the chimeric follicles reported by other groups [22] Our results are in agreement with theirs that stem cells in OSE can generate oocyte-like structures *in vitro *

Virant-Klun et al. [27–29] Putative stem cells with an embryonic character isolated from the ovarian surface epithelium of women with no naturally present follicles and oocytes Parthenogenetic embryo-like structures in the human ovarian surface epithelium cell culture in postmenopausal women with no naturally present follicles and oocytes Stem cells in aged mammalian ovaries	Small (diameter 2–4 *μ*m) round putative stem cells also able to forming oocyte-like cells *in vitro* isolated from human OSE These cells expressed mRNA for pluripotent markers like Oct-4, SSEA-4, Nanog, and Sox-2 After 20 days of culture formed oocyte-like cells expressing VASA, c-KIT and ZP2 transcripts Accompanying bubble-like putative stem cells growing in close contact with oocytes possibly acting like granulosa cells supplying essential cellular machinery to the developing germ cells Oocytes derived from these putative stem cells *in vitro* underwent parthenogenetic activation to form blastocyst-like structures Investigators concluded that they had discovered small cells with pluripotent characteristics comparable to VSELs found in other adult human tissues and organs	Surface epithelial location of the stem-like cells in postmenopausal ovaries reported by them matches initial reports of the location of presumptive GSC (MVH-BrdU double-positive cells) in juvenile and young adult mouse ovaries [7] The cells reported are probably the VSELs

Parte et al. [30] Detection, characterization, and spontaneous differentiation *in vitro* of very small embryonic-like putative stem cells in adult mammalian ovary	Two distinct populations of putative stem cells detected in scraped OSE of adult mammalian ovary, namely, VSELs (1–3 *μ*m) and progenitor stem cells (4–7 *μ*m) termed OGSCs VSELs express nuclear OCT-4 whereas the OGSCs show cytoplasmic OCT-4 Pluripotent markers Oct-4, Oct-4A, Nanog, Sox-2, TERT, and Stat-3 in human and sheep OSE c-KIT, DAZL, GDF-9, VASA, and ZP4 expressing oocyte-like cells spontaneously differentiate in three weeks cultures	VSELs are the quiescent stem cell population that undergo asymmetric cell division whereas the OGSCs are the progenitors similar to A_dark_ SSCs in testis, undergo extensive proliferation, and form aggregates just like cytoplasmic bridges in testis [20] VSELs are totipotent to pluripotent in nature and give rise to OGSCs which further differentiate into oocyte-like structures, parthenotes, neuronal-like cells, and so forth Observed close association of developing oocytes with mesenchymal cells *in vitro* formed by EMT of the OSE cells in initial cultures, similar to the results published recently [41]. We propose that granulosa-like cells are formed by EMT Thus VSELs differentiate to give rise to oocytes whereas the epithelial cells undergo EMT to form supporting granulosa-like cells—thus resulting in primordial follicle assembly

## Abbreviations

BM: Bone marrow
BMT: Bone marrow transplantation
EMT: Epithelial mesenchymal transition
ES: Embryonic stem cells
FGSCs: Female germline stem cells
GFP: Green fluorescent protein
H & E: Hematoxylin & eosin staining
LRCs: Label retaining cells
MSC: Mesenchymal stem cells
MVH: Mouse VASA homolog
Oct-4: Octamer binding protein 4
OGSC: Ovarian germ stem cells
OSE: Ovarian surface epithelium
PB: Peripheral blood
PGC: Primordial germ cells
Q-PCR: Quantitative polymerase chain reaction
RT-PCR: Reverse transcription polymerase chain reaction
SSCs: Spermatogonial stem cells
VSELs: Very small embryonic-like stem cells.
